# Endoscopic reversal of the modified Long vertical gastroplasty

**DOI:** 10.1093/jscr/rjae097

**Published:** 2024-02-29

**Authors:** Talia Abelman, Adam Skidmore

**Affiliations:** Department of General Surgery, Albury Wodonga Health, 201 Borella Road, Albury, NSW 2640, Australia; Department of General Surgery, Albury Wodonga Health, 201 Borella Road, Albury, NSW 2640, Australia

**Keywords:** bariatric surgery, endoscopic, vertical gastroplasty, reversal, minimally invasive

## Abstract

Endoscopic techniques are revolutionizing bariatric surgery because of the significant risks that obesity places on both anaesthesia and surgery. Due to the high number of long-term complications that may be associated with bariatric surgery, and the relative safety of endoscopy compared to operative management, endoscopic methods for reversal of previous bariatric techniques are becoming the favoured approach. We describe the use of an endoscopic stent to assist with endoscopic reversal of a modified Long vertical gastroplasty. The modified Long vertical gastroplasty was a restrictive bariatric procedure that was effective to promote weight loss, but had adverse effects including dysphagia, reflux, indigestion and weight regain. The use of an adjunct stent not only treated the patient’s reflux by dilating the stricture but allowed for erosion of the sutures intra-luminally to be removed endoscopically. The endoscopic reversal of this technique is safe and helped to relieve the patient’s symptoms of reflux and dysphagia.

## Introduction

Bariatric surgery is a long-term solution for obesity that has been described since the 1950s. As the incidence of obesity grows, so does the need for innovative, minimally invasive procedures. Due to the high number of long-term complications that may be associated with bariatric surgery, and the relative safety of endoscopy compared to operative management, endoscopic methods for reversal of previous techniques are becoming the favoured approach. We describe the use of an endoscopic stent to assist with endoscopic reversal of a modified Long vertical gastroplasty.

The modified Long vertical gastroplasty is an open surgical technique that has since been surpassed by vertical gastric banding, laparoscopic sleeve gastrectomy and Roux-en-y gastric bypass.

Early techniques of vertical gastroplasty, described by Long [1] in 1977 involved placing an oblique staple line from the fundus of the stomach to create a stoma along the lesser curvature. This was considered a restrictive technique, with the aim of reducing the size of the stomach so that patients would have earlier satiety and consume less. However, the gastric pouch was able to dilate, so the modified Long vertical gastroplasty [2] involved using a stapler to form a gastric pouch with the addition of nylon sutures placed through the staple line. A 36Fr bougie was passed down the oesophagus, a 90 mm staple gun was then placed at a 90° angle to the Angle of His to create the pouch. To reinforce the pouch and prevent dilatation nylon sutures were placed through the staple line, around the lesser curvature of the stomach and to the anterior surface of the stoma to indent the serosa but not firm enough to compress the bougie. A total of three sutures were placed at 1.2 cm intervals.

The common complications of this procedure, similar to other restrictive bariatric procedures, were weight regain, dysphagia, gastro-oesophageal reflux, gastric outlet stricture or gastro-gastric fistula and may require either re-operation or reversal.

The technique previously described to reverse the Long vertical gastroplasty is a laparoscopic technique in which the sutures are accessed through laparoscopic ports and cut using laparoscopic scissors [3].

We describe the first endoscopic technique to reverse the modified Long vertical gastroplasty.

## The case

Patient X is a 64-year-old female who underwent a Long vertical gastroplasty in 1982. Her original operation was described as above, however, only two nylon sutures were placed compared to three. Her weight prior to the operation was 125 kg (body mass index (BMI) 48.2) and following operative management, her lowest weight was 92 kg (BMI 35.1). Post-operatively, the patient developed severe reflux, indigestion and dysphagia. These symptoms were due to a stricture at the site of the suture line. Over the course of 4 years the patient underwent multiple gastroscopies and dilatations to alleviate symptoms.

Following three previous attempts at endoscopic dilatation using both pneumatic and balloon techniques, the decision was made to place an AXIOS™ 15 mm × 10 mm stent at the suture line.

The stent was effective at reducing the patient’s reflux symptoms, however her dysphagia persisted.

**Figure 1:**
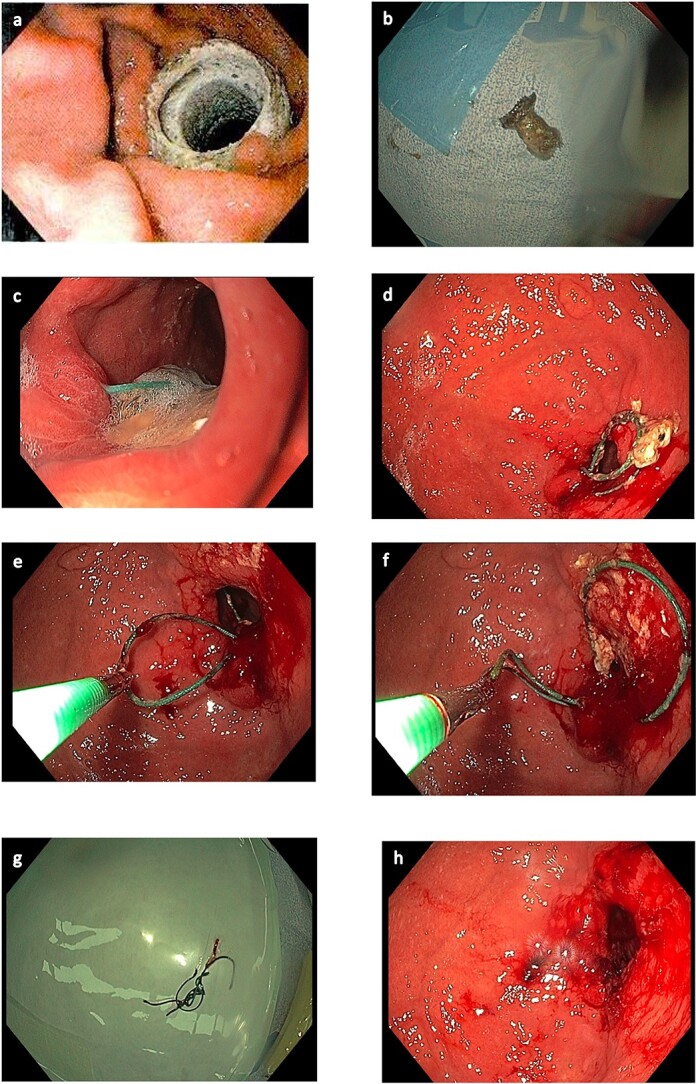
Endoscopic images. (a) AXIOS stent™ in situ. (b) AXIOS stent removed. (c) Sutures eroded, viewed endoscopically. (d) Eroded sutures viewed endoscopically within gastric body. (e) Endoscopic removal of sutures. (f) Endoscopic removal of sutures. (g) Sutures removed. (h) Gastric body following removal of sutures.

Seven months following the original placement of the AXIOS™ stent an Upper GI endoscopy was performed under a general anaesthetic in the supine position. An endo-tracheal tube was placed by the anaesthetic team. A gastroscope was passed from the oesophagus to the second part of the duodenum without difficulty. The gastro-oesophageal junction was at 36 cm from the incisors and a small hiatal hernia was noted. Two Ethibond sutures had eroded through to the lumen and were able to be removed endoscopically by cutting with Endo Scissors. The total procedure time was 60 min. The patient suffered no immediate complications and on review in the outpatient setting 6 weeks following the procedure was recovering well (Fig. 1).

## Discussion

Developing endoscopic techniques for the reversal of bariatric procedures is vital due to the increased risk obesity places on both surgery and anaesthesia. The use of endoscopy has previously been described for the removal of adjustable gastric bands however this is the first endoscopic technique described for the reversal of the modified Long vertical gastroplasty.

The extended placement of an AXIOS™ stent causes pressure necrosis between the stent and the gastric wall, thus allowing for the sutures to erode intra-luminally and to be accessed endoscopically.

The benefits of an endoscopic approach compared to the previously described laparoscopic approach is multi-faceted, particularly within the obese patient group.

Obesity presents greater challenges for the anaesthetic team both when intubating the patient and with on-going ventilation throughout the procedure [4].

Furthermore, central adiposity can cause decreased visualization within the operative field, posing a challenge to laparoscopic procedures [5]. This is particularly evident when attempting to visualize the lesser curvature, as fatty, enlarged livers may be at risk of damage when being retracted to visualize the operating field. The placement of laparoscopic ports also poses a risk to these organs. The use of endoscopy minimizes damage to the gastric serosa and other intra-abdominal organs.

Due to the decreased oxygen tension within obese tissue there is a well-established connection between obesity and wound complications or infections [5]. Surgical incisions, albeit laparoscopic, convey risks of incisional hernia and wound infection. An endoscopic approach avoids the need for any surgical incisions, this not only reduces the length of the procedure but also the risk of wound complications.

When using an endoscopic approach, patients are able to be discharged from hospital the same day and are able to return to regular activities the following day with no restrictions.

A shorter recovery time reduces the risk of deep vein thrombosis, which has an increased incidence within the obese population [5]. Furthermore, quicker recovery time is better from a psychosocial perspective for the patient and reduces hospital costs.

## Conclusion

Endoscopic techniques are revolutionizing bariatric surgery due to the significant risks that obesity places on both anaesthesia and surgery. The use of an adjunct stent not only treated the patient by dilating the stricture but allowed for erosion of the sutures intra-luminally to be removed endoscopically. We describe a safe, effective, endoscopic method for the reversal of a modified Long vertical gastroplasty that reduces recovery time and risk to the patient.
